# Problems and Barriers Related to the Use of AI-Based Clinical Decision Support Systems: Interview Study

**DOI:** 10.2196/63377

**Published:** 2025-02-03

**Authors:** Godwin Denk Giebel, Pascal Raszke, Hartmuth Nowak, Lars Palmowski, Michael Adamzik, Philipp Heinz, Marianne Tokic, Nina Timmesfeld, Frank Brunkhorst, Jürgen Wasem, Nikola Blase

**Affiliations:** 1 Institute for Healthcare Management and Research University of Duisburg-Essen Essen Germany; 2 Department of Anesthesiology Intensive Care and Pain Therapy University Hospital Knappschaftskrankenhaus Bochum Bochum Germany; 3 Center for Artificial Intelligence Medical Informatics and Data Science University Hospital Knappschaftskrankenhaus Bochum Bochum Germany; 4 Knappschaft Kliniken GmbH Recklinghausen Germany; 5 Department of Medical Informatics, Biometry and Epidemiology Ruhr University Bochum Bochum Germany; 6 German Sepsis Society Jena Germany

**Keywords:** decision support, artificial intelligence, machine learning, clinical decision support system, digitalization, health care, technology, innovation, semistructured interview, qualitative, quality assurance, web-based, digital health, health informatics

## Abstract

**Background:**

Digitalization is currently revolutionizing health care worldwide. A promising technology in this context is artificial intelligence (AI). The application of AI can support health care providers in their daily work in various ways. The integration of AI is particularly promising in clinical decision support systems (CDSSs). While the opportunities of this technology are numerous, the problems should not be overlooked.

**Objective:**

This study aimed to identify challenges and barriers in the context of AI-based CDSSs from the perspectives of experts across various disciplines.

**Methods:**

Semistructured expert interviews were conducted with different stakeholders. These included representatives of patients, physicians and caregivers, developers of AI-based CDSSs, researchers (studying AI in health care and social and health law), quality management and quality assurance representatives, a representative of an ethics committee, a representative of a health insurance fund, and medical product consultants. The interviews took place on the web and were recorded, transcribed, and subsequently subjected to a qualitative content analysis based on the method by Kuckartz. The analysis was conducted using MAXQDA software. Initially, the problems were separated into “general,” “development,” and “clinical use.” Finally, a workshop within the project consortium served to systematize the identified problems.

**Results:**

A total of 15 expert interviews were conducted, and 309 expert statements with reference to problems and barriers in the context of AI-based CDSSs were identified. These emerged in 7 problem categories: technology (46/309, 14.9%), data (59/309, 19.1%), user (102/309, 33%), studies (17/309, 5.5%), ethics (20/309, 6.5%), law (33/309, 10.7%), and general (32/309, 10.4%). The problem categories were further divided into problem areas, which in turn comprised the respective problems.

**Conclusions:**

A large number of problems and barriers were identified in the context of AI-based CDSSs. These can be systematized according to the point at which they occur (“general,” “development,” and “clinical use”) or according to the problem category (“technology,” “data,” “user,” “studies,” “ethics,” “law,” and “general”). The problems identified in this work should be further investigated. They can be used as a basis for deriving solutions to optimize development, acceptance, and use of AI-based CDSSs.

**International Registered Report Identifier (IRRID):**

RR2-10.2196/preprints.62704

## Introduction

### Background

AI, and in particular machine learning (ML), is becoming more prevalent in health care systems worldwide. This is illustrated, for example, by the number of AI- or ML-enabled medical devices approved in the United States. Here, the annual increase in 2020, 2021, and 2022 was 39%, 15%, and 14%, respectively, and was forecasted to rise to ≥30% in 2023 [1]. In January 2024, the Food and Drug Administration listed 692 medical devices enabling AI and ML [1]. For Europe, no reliable statement can be made about the number of approved AI- or ML-enabled devices as the corresponding European database, the European Database on Medical Devices, does not provide a corresponding filter function [2]. This means that it is not possible to search explicitly for devices with AI or ML component.

AI-enabled devices cover a wide range of medical specialties, such as dermatology, diabetology, gastroenterology, gynecology, heart surgery, cardiology, nephrology, ophthalmology, orthopedics, pathology, psychiatry, and radiation oncology [3]. In the United States, in the first 7 months of 2023, a total of 79% of newly approved devices covered the field of radiology; 9% covered the cardiovascular field; 5% covered the field of neurology; 4% covered the field of gastroenterology or urology; 2% covered the field of anesthesiology; and 1% each covered the ear, nose, and throat as well as ophthalmic fields [1].

Analogous to the large number of medical specialties using AI, there are also many different types of applications. For example, AI can support the detection of infectious disease outbreaks; identify rare and common diseases by combining clinical, genetic, and many other laboratory test results; and support hospital business operations [4].

In addition to the various possibilities to benefit from AI in health care, there are also some concerns and problems with its use. These can be categorized in different ways. While Farhud and Zokaei [5], for example, identified “privacy and data protection,” “informed consent and autonomy,” “social gaps and justice,” and “medical consultation, empathy, and sympathy” as problem areas, Khan et al [6] identified “data collection and algorithm developing,” “ethical,” “social,” and “clinical implementation” as concerns.

An interesting application of AI in the medical context is its use in CDSSs. In general, CDSSs can be classified into knowledge based or non–knowledge based. While the knowledge-based systems follow clear rules (if-then statements), the non–knowledge-based systems use AI, ML, or statistical pattern recognition to make decisions on a data source [7]. Several approaches exist for ML. These include supervised learning, where algorithms benefit from information provided and labeled by humans (eg, by adding the final diagnosis to a set of clinical data), and unsupervised learning, where algorithms search for patterns in the underlying data on their own [8]. There is also reinforcement learning, where algorithms learn by interacting with an environment and receive feedback in the form of rewards to adapt and improve their actions [9]. AI-based CDSSs can be further distinguished based on the number of characteristics (eg, support on demand or unprompted) and in terms of their functions (eg, supporting diagnosis, outcome prediction, treatment planning, prescribing and managing medications, preventative care, chronic disease management, image interpretation, and many others) [10].

Regardless of AI, CDSSs also have several problems in addition to their many benefits. These problems include their content (“elimination or shifting of human roles,” “difficulty in keeping content current,” and “inappropriate content”) and their presentation (“rigidity of the system,” “alert fatigue,” and “potential for errors”) [11].

AI has the ability to evaluate large amounts of data where humans would fail because they cannot perceive the entire context beyond the data [12]. Thus, clinical decision-making can benefit from the use of AI. It can assist with the prognosis, diagnosis, and treatment of diseases; support the clinical workflow; and expand the availability of medical expertise [13]. However, in addition to the benefits, the problems and barriers of AI applications should be critically and responsibly considered rather than ignored [3].

### Objectives

The use of AI-based CDSSs affects a large number of different stakeholders, such as physicians, caregivers, patients, and developers, and raises a multitude of different questions (eg, of legal or ethical nature). Therefore, in this research, various stakeholders were interviewed with the aim to identify potential problems and barriers related to AI-based CDSSs.

## Methods

### Overview

Qualitative research, concretely, semistructured expert interviews, was conducted to identify problems and barriers in the context of AI-based CDSSs. The decision to conduct qualitative research was made with the aim to approach the question exploratively because the use of AI is still not well established in health care systems worldwide. To ensure transparency in all aspects of our qualitative research, we followed the standards by O’Brien et al [14] and subsequently checked the manuscript by applying the 32-item COREQ (Consolidated Criteria for Reporting Qualitative Research) checklist [15].

### Theoretical Framework

This study is part of a larger research project called KI@work (User-Oriented Requirements for AI-Based Clinical Decision Support Systems) funded by the German Federal Joint Committee. Building on the results of this interview study, a corresponding scoping review, and focus groups with physicians and carers, a quantitative survey of physicians will be developed to investigate their preferences as well as problems in the context of AI-based CDSSs.

The aim was to collect the perspectives and opinions of experts. The interviews were conducted with stakeholders playing a pivotal role in the context of AI in medicine. On the basis of a previously conducted scoping review in the context of the project, a guideline was created and used to structure the interviews.

During the interviews, we asked about both problems and barriers as well as solutions to overcome them. This paper focuses especially on the problems and barriers identified in the underlying conversations.

### Participant Selection

Before contacting potential participants, the project consortium discussed relevant stakeholders. During the discussion, they came to the consensus that, in addition to representatives of patients, service providers, AI developers, and health insurance funds, further experts with knowledge on regulation of AI as well as experts on ethical questions should be included.

Once the relevant stakeholder groups were determined, the University of Duisburg-Essen recruited corresponding experts with the help of the entire consortium. Before conducting the interviews, the stakeholders received an informative letter about the topic of the interviews—problems in the implementation of AI-based CDSSs in patient care—and further information about the procedure, including information about data protection, via email.

A total of 4 contacted stakeholders did not respond to the request. There was no clear refusal to participate in the interviews. After recruitment, during the interviews, none of the experts included dropped out of the study.

### Setting and Data Collection

All the interviews were held online either via the videoconferencing platform integrated into Microsoft Teams or Zoom (Zoom Video Communications). Each expert interview was guided by a moderator and accompanied by at least one additional comoderator. All 3 moderators (GDG, PR, and NB) were from the Institute for Health Care Management and Research, University of Duisburg-Essen. While one moderator (NB) is head of the research team, the 2 others are scientific staff within the team. In total, 2 moderators were male (GDG and PR), and one moderator was female (NB). Of the moderators, 2 have a (health) economic background, and the third moderator holds a medical doctor degree in addition (NB). GDG and NB are experienced in conducting qualitative research, especially interviews. PR gained experience in conducting interviews through his previous work in market research. There were no relationships between the researchers and the interviewees. The researchers did not have a clear stance either for or against the use of AI in health care. No further circumstances or events influencing the conversations are known.

Preliminary results of a scoping review conducted within the project (to be published) before the stakeholder interviews served to develop a noninfluencing interview guideline. This guideline followed a uniform structure, including three main topics: (1) general problems with AI-based CDSSs, (2) problems in the development process of AI-based CDSSs, and (3) problems in the clinical use of AI-based CDSSs. Each of the topics started with open questions followed by more specific questions. Each topic included a core set of fixed questions for all stakeholders and was also slightly extended or adapted to the stakeholders to receive stakeholder-specific information (Multimedia Appendix 1).

Data collection took place between the beginning of June 2023 and the middle of August 2023. If the interviews were conducted using Microsoft Teams, they were automatically transcribed using the integrated function. The machine-generated transcripts were revised and pseudonymized by research assistants. If the interviews were conducted using Zoom, the recordings were transcribed by research assistants. In both cases, the transcripts were quality checked by 1 of the 3 moderators. The quality-assured transcripts were sent to the corresponding stakeholders interviewed to allow for requests for changes within 1 week (if no request has been made to extend the deadline). During the study, no adjustments (except for the stakeholder-specific questions in the guideline) were made to the method of data generation as the interviews worked without problems and the guideline proved to be understandable.

### Data Analysis

After incorporating the requested changes by 3 stakeholders, the transcripts were loaded into MAXQDA (VERBI Software GmbH), and video recordings were deleted. Subsequently, data analysis started in September 2023 and was completed in October 2023.

As recommended by Kuckartz [16], deductive codes were defined before data analysis and applied subsequently to the transcripts (GDG). Deductive codes were determined according to the structure of the interview and included the 3 topics of the guideline (“general,” “development,” and “clinical use”).

The statements identified were concisely summarized and printed on individual pieces of paper (GDG). In a subsequent workshop (GDG, NB, and PR), the printed statements that were thematically similar were grouped together into clusters (“problem areas”). Whenever a problem or barrier arose that did not fit into an existing cluster, a new cluster was created. The clusters were named appropriately according to the problems and barriers they contained.

Subsequently, thematically similar clusters were organized into higher-level groups (“problem categories”) and named accordingly. Finally, the stakeholder statements and their respective summaries were sorted into a matrix with deductive codes (“general,” “development,” and “clinical use”) on the horizontal axis and inductive codes (“problem categories” and “problem areas”) on the vertical axis.

### Ethical Considerations

As the topic of the interviews was not a sensitive subject and sociodemographic or other potentially identifying characteristics of the interviewees were not collected, there was no need for approval by an ethics review board. This was also confirmed by the Ethics Committee of the Faculty of Medicine of the University of Duisburg-Essen. During the introduction, before the interviews, the participants were informed about the exact procedure and conditions and asked for their consent to participate. Through pseudonymization and deletion of the recordings, reassignment of statements to participants was no longer possible. On request, the experts received an expense allowance of €120 (US $124.27) for their participation. In the end, 4 of the 15 interview partners did so.

## Results

### Overview

A total of 15 interviews were conducted with 17 experts. In total, 13% (2/15) of the interviews conducted were double interviews with 2 interview partners in each. Experts included all requested stakeholder groups: a patient and a physician representative, developers of AI systems, consultants, experts in the fields of quality assurance and quality management, caregiver representatives, members of research institutions in the fields of AI and health care as well as social and health law, a representative of a health insurance fund, and an expert from an ethics committee. In general, intrinsic motivation and participation were high. An overview of each interview is provided in Table 1.

**Table 1:** Conducted interviews.

Number	Stakeholder	Setting	Date
1	Caregiver representative	Individual interview	June 5, 2023
2	Quality management representative	Individual interview	June 5, 2023
3	Researcher—AI^a^ in health care	Individual interview	June 6, 2023
4	Medical product consultant	Individual interview	June 16, 2023
5	Caregiver representative	Individual interview	June 27, 2023
6	Caregiver representative	Double interview	June 27, 2023
7	Quality assurance representative	Double interview	July 4, 2023
8	Representative of an ethics committee	Individual interview	July 17, 2023
9	Researcher—social and health law	Individual interview	July 19, 2023
10	Medical product consultant	Individual interview	July 27, 2023
11	Developer of AI-based CDSSs^b^	Individual interview	July 27, 2023
12	Patient representative	Individual interview	July 31, 2023
13	Physician representative (intensive care)	Individual interview	August 8, 2023
14	Developer of AI-based CDSSs	Individual interview	August 11, 2023
15	Representative of a health insurance fund	Individual interview	August 17, 2023

^a^AI: artificial intelligence.

^b^CDSS: clinical decision support system.

**Figure 1:**
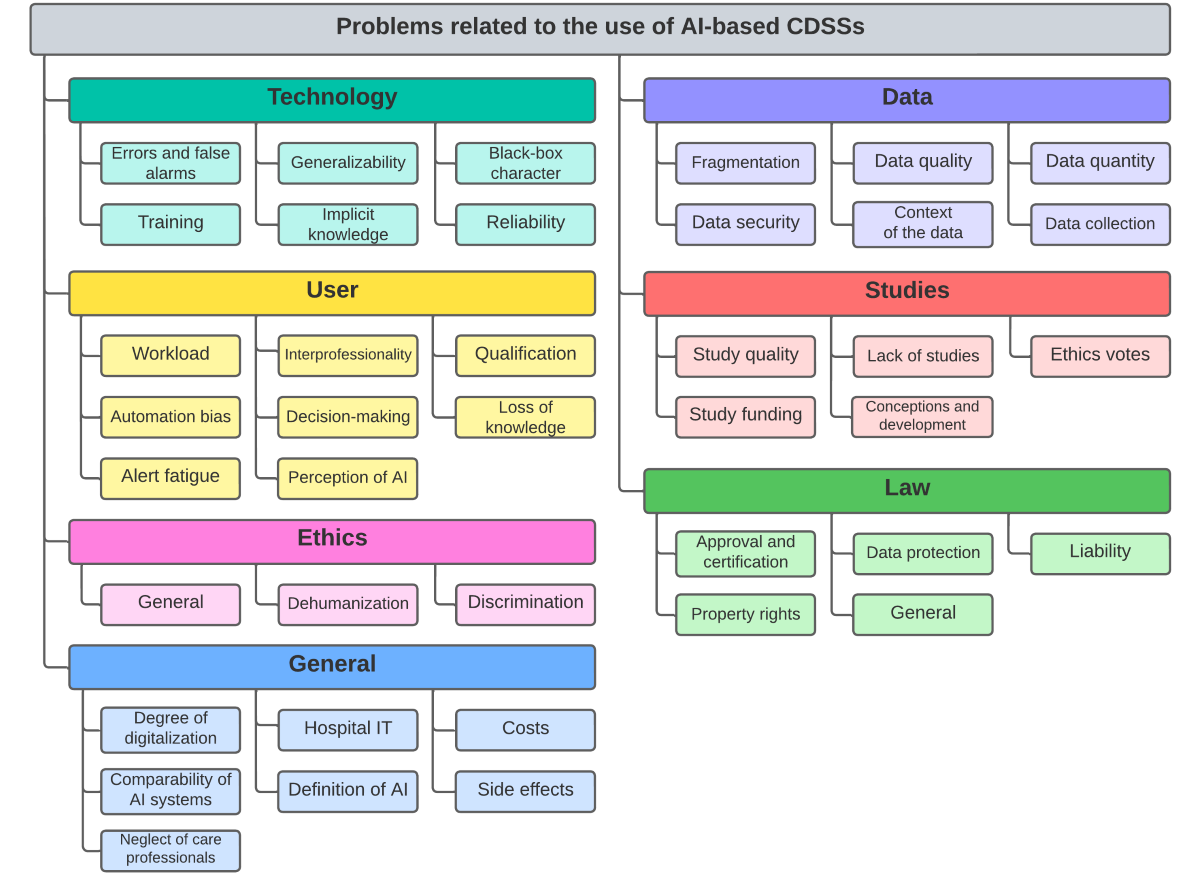
Identified problem categories and corresponding problem areas. AI: artificial intelligence; CDSS: clinical decision support system.

The interviews provided 309 statements on problems and barriers in the context of AI-based CDSSs. A total of 7 problem categories were identified: “technology,” “data,” “user,” “studies,” “ethics,” “law,” and “general.” The categories with their corresponding problem areas are shown in Figure 1.

Even if the aim of qualitative research is more explorative than deterministic, it should be mentioned that some problem categories were more often thematized than others (user: 102/309, 33%; data: 59/309, 19.1%; technology: 46/309, 14.9%; law: 33/309, 10.7%; general: 32/309, 10.4%; ethics: 20/309, 6.5%; studies: 17/309, 5.5%). The problem categories are described in the following sections, and relevant expert statements are reproduced to illustrate the problems. The order of the problem categories does not indicate their relevance. A final list with the systematization of all identified problems can be found in Multimedia Appendix 2.

### Technology

Problems described in the “technology” category were directly related to the technology underlying AI-based CDSSs. Named problems concerned 6 areas: “errors and false alarms,” “generalizability,” “black-box-character,” “training,” “implicit knowledge,” and “reliability.”

“Errors and false alarms” were observed in clinical use and in general. While incorrect recommendations resulting from use were seen as a threat to patients, a more general problem in this context was the tension between sensitivity and specificity:

[W]ith every AI procedure...there exists an area of conflict. So do I tend to develop the system more towards the direction of being more sensible, so to speak. In other words,...if it triggers, there’s a high probability that it’s actually correct. But then you lose, then suddenly there might be cases that you don’t diagnose....

Developer of AI-based CDSSs

In addition to weighing up individualization and generalization during the development process and the system adaptation to local data before use, named problems with “generalizability” were of a more general nature. These problems were observed particularly in relation to regional validity, hospital-specific validity (eg, specialized or smaller hospitals), and population-based validity (eg, genetic). As AI is always developed on a distinct database, experts questioned whether the systems were ubiquitously valid. One expert described that the quality of the results in the real setting is not comparable with the quality of the results based on training and test data:

Yes, I see problems with generalizability. We hear from several developers that if they have a data set of a hundred thousand patients, take 50,000 for training and test it with the other 50,000, then they get good results. If they take data outside of this cohort, the results are different.

Medical product consultant

Furthermore, the “black-box-character” problem was thematized. In general, more complex AI systems tend to be not comprehensible to humans. As a result, users have a lack of understanding of AI-proposed decisions. This is not only a general problem but also a concrete problem during clinical use, as described by a caregiver representative:

[T]raceability is a very important factor for such a system; if I, as a user or collaborator, don’t understand it, then the collaboration doesn’t work well either.

Caregiver representative

During the development process, problems can also occur in the “training” of the AI. This is the case if the training is suboptimal or even poor and biased. This might be due to (1) an insufficiently analyzed database, (2) biased training because of too specific datasets, or (3) the system learning from lowly qualified users. Another problem was observed if the development phase was not completed before actual clinical use (eg, due to the prospect of quick profits). If the development phase is not supervised, so-called drifts can emerge, and the validity of systems might change over time.

Further problems were expressed in the context of “implicit knowledge.” As this type of knowledge cannot be represented in words or numbers, experts explained that it cannot be integrated into AI-based CDSSs. Problems in this context concerned mainly clinical use because experts stated the need for this knowledge and warned against neglecting it by using AI. Furthermore, it was assumed that the lack of implicit knowledge might lead to a reductionist patient image:

We don’t have the context that doctors sometimes have when the patient is right in front of them, and they see him as a human being and ask a few questions. So that’s why it’s very dangerous, because we end up with a very reductionist image of a patient as far as the AI system is concerned.

Developer of AI-based CDSSs

“Reliability” was the last problem area thematized within “technology.” A general problem leading to lack of reliability was observed in the functionality of AI, which is based on probabilities and statistics. According to one expert, these would not always lead to the right decisions:

[T]he problem with AI is that it is not a physical algorithm, but statistics. That means there will always be things where you can be wrong.

Medical product consultant

Furthermore, such systems would neither critically question inputs nor provide absolute reliability. Premature deployment in resource-critical care situations was seen as a particular risk.

### Data

“Data” as the underlying input of AI-based CDSSs are essential for the development and use of these systems. During the interviews, numerous problems were described in the context of data. These included “fragmentation,” “data quality,” “data quantity,” “data security,” “context of the data,” and “data collection.”

The first problem in this category is the “fragmentation” of the health care system and, subsequently, the fragmentation of data. Problems concerning “fragmentation” usually have a general background but have a strong impact on the development process. In particular, the German health care system is characterized by a great variety of institutions and actors with different interests, making it very complex. Breaks exist at the boundaries between different sectors (eg, between inpatient and outpatient supply), which means that data are not collected uniformly and, therefore, are often not comparable. In some cases, there was a lack of uniform standards. These problems are also reflected in the development process of AI-based CDSSs. Nonharmonized, nonunified, and unprocessed data, as well as a multitude of different actors, lead to major barriers for the development of these systems. One expert described a lack of standardization as a problem that hinders the comparability of data:

Well, first of all, I would say that it heavily relies on the training data, that we have different standards there. And the question is to what extent they can be harmonized or standardized, so that you’re not comparing apples with oranges....

Representative of an ethics committee

Once the data had been collected, their quality was described to be often insufficient. It was even stated that data do not always represent reality. The insufficient “data quality” is especially a problem during the development process. In this context, the “garbage-in, garbage-out” principle was mentioned. It states that, if the data entered are not good, the results will not be good either:

Because that’s the garbage-in, garbage-out principle; the worse the data you put in, the worse the result will be.

Developer of AI-based CDSSs

A threat in the context of low-quality data was observed in the use of suboptimal data if no other data exist. This might lead to the development of an AI-based CDSS that produces wrong results and is subsequently inappropriate for clinical use.

Experts also saw a problem in “data quantity.” While, on the one hand, there was a general lack of data, experts such as a researcher in the field of AI in health care mentioned especially a lack of structured and quality-proved data on the other hand:

I think there is a quality problem with the data, that’s undisputed....there is a massive problem, but our main problem is the quantity of data that is available at all.

Researcher—AI in health care

Furthermore, it was mentioned that some data are not available in digitized form. A special case was observed in rare treatment situations as these would not provide a sufficient data basis. In contrast to the lack of data, one developer mentioned an uncontrolled flood of data.

“Data security” was reported as a further problem. In general, the experts themselves questioned data security and mentioned that patients are particularly afraid of data leaks, too. According to the experts, those leaks and problems with the IT and its cybersecurity could emerge during the clinical use of AI-based CDSSs. A threat was observed in hacker attacks and subsequent manipulation or deletion of training data or algorithms, as well as system failures. The risk of being affected by hacker attacks and the absolute danger that this poses were described as being increased in the context of AI:

Data protection is certainly a problem, the more AI you have, the more you are likely to be affected by external hacker attacks and so on. So, because if I have access to the system, the consequences of a hacker attack will be on another level.

Representative of a health insurance fund

A problem in clinical use could emerge if the (medical) “context of the data” changes. Therefore, the quality of the information provided by AI-based CDSSs could change. An example given by an expert of such an altering context was a changing understanding of diseases or a change in diagnoses over time:

So, you can’t just say that this is a technical problem. You always have to ask again about the current state of science. Is it still valid if I take the data from eight or ten years ago, because sometimes the concept of illness changes, e.g. in the case of psychological experiences, but also in the case of other diagnoses.

Quality assurance representative

Finally, it was explained that the “data collection” process might be a problem. Experts stated that only a fraction of treatment data is available in digital form and that the extraction of analog data can be very complex.

### User

A major problem category was the “user” of AI-based CDSSs. Related problem areas were “workload,” “interprofessionality,” “qualification,” “automation bias,” “decision-making,” “loss of knowledge,” “alarm fatigue,” and “perception of AI.”

Lack of time is omnipresent among physicians and caregivers. Therefore, experts warned of additional time expenditure, especially in the context of clinical use. Nevertheless, the lack of time and the additional workload were also a problem during development because caregivers were therefore not able to insert data for training purposes. In the context of clinical use, an additional workload was stated, assumed, considered, or feared by different experts:

But in cases where someone has to enter something, you must be careful, what does the doctor have to do, are you taking time away from the patient again?

Quality assurance representative

Experts stated that some data cannot be collected automatically and, thus, would lead to a need for additional personnel. Potentially necessary manual corrections of incorrect measurements would be required. Furthermore, a problem related to workload was the attempt to misuse AI-based CDSSs to cut staff costs.

A problem area concerning development was “interprofessionality.” Generally, experts stated the need for interdisciplinary development of an AI-based CDSS. Otherwise, they foresaw problems in clinical use or even stated that, without interdisciplinary development, no useful systems would be developed at all. In particular, the lack of involvement of medical and care professionals was expressed by the experts. They mentioned that medical professionals are more likely to notice professional errors during development but also that they tend to underestimate the effort to develop an AI-based CDSS. A further problem with the involvement of physicians is that the development of an AI-based CDSS is usually only a secondary activity for them. The caregiver representatives felt that caregivers are insufficiently involved in the development of such systems, and thereby, important caregiver knowledge would not be incorporated. Development problems are also aggravated by the lack of skilled workers with knowledge of AI.

Once started, development can be difficult because of nonfunctioning cooperation between different professions, the time required for interprofessional communication, different specialist terminology, and conflicts of interest between actors involved:

Yes, so I would totally agree. So that’s clear in such a—especially in the development phase, there are various stakeholders and various interests that simply come together, and to even get into the discourse, to have a conversation and to unite them, I think that’s a very high art.

Caregiver representative

The “qualification” of users can also be a problem. It was stated by the experts that, in general, the users are poorly qualified for the use of AI-based CDSSs. They sometimes lack digital experience or further training. A caregiver representative mentioned a low intrinsic motivation of care professionals to deal with the topic of AI-based CDSSs. A lack of experience might also lead to more difficult interpretation and communication in the context of the systems. One expert explained that a lack of training was a problem and that it was not enough to train just 1 employee. Otherwise, it was likely that further problems would result:

I think one problem is the training. No matter who uses it, I must train these people. And I believe that’s always a source of error, so it’s shown to one person. The entire staff that has to use it simply isn’t thoroughly trained. Then mistakes can of course happen in the application of this AI.

Representative of a health insurance fund

Experts described that users tend to prefer suggestions from (AI-based) CDSSs and ignore contradictory information provided without automation even if it is correct. This was described as blind trust, blind replying, uncritical adoption, or “automation bias.” It was assumed that automation bias can lead to inadequate action and potentially jeopardize patient health. The problem was reinforced due to time pressure, stress, and a high shortage of skilled workers. This was especially emphasized by a caregiver representative:

So, I definitely see the danger that, especially against the background of such a fast clinical routine, the recommendation is simply adopted without being asked.

Caregiver representative

In addition to automation bias, further problems exist within the “medical or caregiving decision-making” process. Thus, AI-based CDSSs can be seen as a decision-making barrier in the case of disagreements. They might endanger the autonomy of users or undermine the medical and nursing expertise. An expert described this new knowledge asymmetry in which the patient is ahead of the physician as follows:

And I believe that quite a lot of doctors, and certainly a few nursing experts and nurses, see in it a bit of a curtailment of their field, their expertise, their unique selling point. I mean, when AI-based applications are brought into the public, I, as a person who may even be suffering from a rare disease and knows more through these support systems than my treating doctor, then they have to deal with that too, yes.

Quality management representative

A further problem area expressed was “loss of knowledge.” In particular, the risk that the regular use of the systems would result in users no longer being able to access their own knowledge, which would then diminish, was described:

I see the risk of knowledge loss equally significant in all areas, so we’re handing something over to AI in all aspects of life. And we are also losing competencies as a result.

Caregiver representative

Furthermore, experts explained that too many (false) alarms might lead to “alert fatigue.” This means that system users deliberately ignore alerts or no longer manage to follow them up. It was described that the number of alerts produced across all software systems would be overwhelming and lead to alert fatigue:

That’s a huge problem, especially false alarms, regardless of artificial intelligence. I mean, all of our or many of our software systems produce alerts. But in such a volume that one can’t even follow up on them anymore, and then, of course, we’re also talking about alert fatigue.

Quality management representative

Finally, the “perception of AI” was described as a problem area emerging from the user side. The problem partly originated in negative communication. The perception described ranged from low or lack of trust to doubts about validity and benefit to a partly irrational fear. A negative perception was observed among patients and health care providers but also among authorities and notified bodies. Health care providers described a fear of being replaced by AI. Excessive expectations of AI and a lack of awareness of the limits of AI systems were also reported. One expert described the perceptions in both directions (positive or negative) as extreme.

In clinical use, it was observed that distrust among users might emerge due to an undermining of medical competence or due to measuring physicians’ diagnostic quality. A lack of trust was associated with a lack of added value provided by the systems. During clinical use, the perception of patients toward AI-based CDSS might also be impacted. This could happen through perceived false alerts or misleading medical wording. One expert mentioned that a big problem lies in failing to open up to AI and focusing too narrowly on its problems:

And I think, that’s one of the biggest problems with AI-based solutions, if I don’t approach the whole issue decisively or openly. That means, if I only see all the risks, if I’m somehow maybe even fear the AI-powered, self-evolving robot that might wipe out humanity, so I’m exaggerating it to some extent, but there are still some people out there who have this idea.

Medical product consultant

### Studies

A further problem category was “studies,” which are necessary to examine and demonstrate the evidence in terms of clinical effectiveness, reliability, and real-world applicability of AI-based CDSSs. This included the “study quality”; a “lack of studies”; and problems with “ethics votes,” “study funding,” and “conception and development.”

In general, experts questioned the quality of available studies. They criticized that studies are not conducted with conscientiousness and that results may be manipulated. Furthermore, a risk of low-quality evidence in studies was described. An expert described the varying quality of studies that must be considered:

That’s just—and I believe that often, work is not done properly enough, because people simply think, “Oh, it’s just a study, and it’ll be fine.” If we look closely...we often find that, well, not all studies are created equally right.

Representative of a health insurance fund

In addition to the quality, the “lack of studies” was expressed to be a problem. It was mentioned that there are only few study results from Germany available. In particular, a lack of valid and reliable studies, prospective studies, and studies on the additional benefits of the systems was named:

The problem with all these AI algorithms so far is that they are based on retrospective data. And what we need is a prospective—or prospective studies with algorithms, to prove that there is actually a prospective advantage compared to standard treatment.

Physician representative—intensive care

“Ethic votes” were described as being an obstacle to study conduct. In detail, according to an expert, this means that ethic votes are usually necessary but might be rejected:

[A]nd even if we had data, we would need appropriate ethics approvals for the data, unless it is de-identified, which simply takes a very long time. And I have actually heard from colleagues that such ethics approvals have been rejected because they were supposed to be used for technical AI studies.

Researcher—AI in health care

A further problem area in the context of studies was “study funding.” The experts called for more funding to be able to conduct studies to prove the evidence of AI-based CDSSs. This was described by a physician representative as giving up medical success due to lack of study funding for fear of AI.

Finally, problems were mentioned in the context of “conception and development.” A threat was observed if developers were not independent of influence. Experts questioned the objectivity and knowledge base of algorithms and the transparency and trustworthiness of the development process. The latter was partly described as exploratory instead of purposeful, and the lack of a holistic view of the data was criticized. This was illustrated with the help of a metaphor by an expert:

So, if you collect data for the sake of data and then run it through a system, with some machine learning algorithm, and then get something nice out of it, it’s just like knowing: Somewhere in a pond there are 20,000 different species of fish. I throw my fishing rod in there, catch a carp and afterwards I end up saying, that’s exactly what I wanted. So maybe we should also think about what kind of data do I need for what, so what are my outcomes....

Caregiver representative

### Ethics

The problem category “ethics” included “general,” “dehumanization,” and “discrimination.” While “general” only consisted of general concerns about ethical problems, the other problem areas were more specified.

During clinical use, “dehumanization” of patients was feared. This was due to the dehumanization of the medical treatment and the risk of deteriorating communication with patients. Experts described that patients might be perceived as data points and their individual stories might not be considered:

But the question is, how can you link this in a meaningful way with general practitioners who actually see the patient and their history and not just specific data points?

Representative of an ethics committee

The most discussed problem area in the context of ethics was “discrimination.” These problems occurred within the “development” category, concerned clinical use, and were partly general. General problems were observed in the reidentification of small population groups and a special paradox:

I see it as a bit of a paradox that, on the one hand, you of course want to collect data from as many minorities as possible to make the data more representative and, on the other hand, you have to classify them and that this can lead to new forms of stigmatization.

Representative of an ethics committee

An inherent problem of the development of an AI-based CDSS was observed in the dependence on the underlying data. The latter were regularly discriminatory or at least imbalanced between different patient groups. One such discrimination was observed in primarily male-oriented data. Once discrimination exists within the data, the AI developed adopts these patterns and reflects them in clinical use. Thus, a threat was described that the use of AI-based CDSSs might result in discrimination, for example, in terms of social inequalities or racist behavior. That this problem is already subject to research was described by a medical product consultant:

Basically, I know that there are a lot of AI research activities to see whether AI behaves in a racist way. So, there are definitely some research projects that point in that direction.

Medical product consultant

### Law

In terms of law-related problems, experts named the problem areas “approval and certification,” “data protection,” “liability,” “property rights,” and “general.” The latter encompassed that AI-based CDSSs are currently not sufficiently reflected in medical law.

In the context of “approval and certification,” 2 problems were described. First, the certification procedure for AI-based CDSSs was described as overly complex and costly. Among other things, additional work due to different requirements for different authorization regions was named. Especially for start-ups with scarce resources, the framework conditions were described as too cost intensive. Second, experts stated that there are regulatory issues in the context of medical device authorization and uncertainties with and an insufficient number of certifying bodies:

The regulator hurdle is the certification as a medical device. There are currently only four notified bodies in the EU for the certification of software as a medical device. And that means we have waiting lists.

Medical product consultant

“Data protection” was found to be a general problem but also a problem during clinical use. For clinical use, on the one hand, there were experts that stated that data protection was a real problem. On the other hand, experts stated the problem that data protection was used as a pretext against the use of AI-based CDSSs or to avoid renewal. Real data protection problems in clinical use were observed, for example, in insufficient reading and writing permissions in the digital patient file for caregivers due to data protection.

In general, requirements for data protection and data security were described as cumbersome, and their implementation was seen as problematic. In particular, data that cannot be anonymized and automated data processing were seen as special issues. To some extent, experts described that data protection would hamper the exchange of data even within the same hospital and would lead to poorer data. In the case of withdrawal of consent to use data, identification of specific data among vast amounts of data might be a problem. Finally, it was criticized that data protection was prioritized over health protection:

[M]y view is that we have a big problem when it comes to data protection. In my opinion, in Germany or at least in my perception, data protection is more important than health protection.

Quality management representative

Furthermore, experts saw problems with “liability.” There were concerns that physicians were always liable for decisions even if the errors stemmed from AI-based CDSSs. It was stated that the problem of liability is a difficult question and that, in some cases, responsibility remains unclear or is not regulated at all:

Is it actually the user or is it the person who developed the system? Who is actually responsible afterwards if there is some damage because the system did not function properly?

Researcher—social and health law

Finally, the issue of “property rights” was raised. Given the multidisciplinary development processes of AI-based CDSSs, it is not always clear who has sovereignty over data and development results. Therefore, conflicts over ownership of data and patents might arise.

### General

The last problem category was “general.” It included all problems that could not be matched to another category. It consisted of (nonlegal) framework conditions such as “degree of digitalization,” “hospital IT,” “definition of AI,” or “costs” on the one hand and of more direct problem areas such as “comparability of AI systems,” “side effects,” or “neglect of caring professions” on the other hand.

Different experts described that there was a lack of digitalization or that the “degree of digitalization” in the health care sector was too low. Furthermore, a lack of integration and, subsequently, a lack of data availability between sectors and institutions was described:

If you look at the overall process that a patient goes through, it is ultimately still too analogue and too disruptive and not digitized enough. That’s a big problem overall as a basis for AI-based support systems. Because ultimately, you need a relatively large amount of data and also from a specific patient in order to compare it accordingly.

Physician representative

A problem area in clinical use was the “hospital IT.” It was described as inadequate and lacking in technical equipment, its reliability was questioned, and a lack of IT staff was mentioned. A lack of system compatibility; a lack of interfaces between individual departments; and the unavailability of standardized, structured data within the hospital were more concrete problems. Furthermore, it was described that hospitals are not considered attractive places to work for IT specialists and that their pay would be too low. The unattractiveness of hospitals for IT specialists was, for example, described by a researcher in the field of AI in health care:

The costs are exploding of course. The IT sins have of course piled up everywhere and you also know that hospitals are not seen as desirable workplaces for IT specialists.

Researcher—AI in health care

Furthermore, problems with “costs” were observed during development in the lack of financial resources for projects, as well as the implementation costs being a barrier for clinical use. A problem that prevents cost savings was observed in AI-based CDSS manufacturers aiming to maximize profits. Within the remuneration system, the lack of specific billing codes for algorithm-based laboratory test results and the absence of separate billing options for AI systems were criticized:

Then we certainly have another issue, that the costs for these systems are currently not separately billable. Instead, they are part of the medical service, and you can take the position, well, that’s just how it is, because the reimbursement code exists for the image and the diagnosis, and if the doctor buys additional software to save time in diagnosis, then that’s an economic effect on his side, which the system doesn’t have to worry about.

Medical product consultant

A challenge in deciding between different AI-based CDSSs, or whether to adopt such a system at all, could arise from the insufficient “comparability of AI systems.” In addition, the lack of reasonable data to make health economic decisions was criticized:

I’m not sure whether those who are supposed to use it have sufficient criteria to decide which system to buy and how to compare the different systems with each other. So how do the different systems on the market actually compete with each other in terms of quality?

Medical product consultant

A problem expressed by caregiver representatives was a “neglect of caring professions” within the context of AI-based CDSSs. A lack of funding for the development of AI in the care sector, as well as a strong focus on medical applications resulting in a deficit in nursing applications, was described.

Furthermore, the occurrence of “side effects” in the clinical use of AI-based CDSSs was named as a problem requiring evaluation and follow-up to guarantee patient safety.

Finally, experts thematized different “definitions of AI” as a problem:

So, if you tell someone in practice that this is artificial intelligence, then there are an incredible number of opinions and ideas about what it could actually be.

Caregiver representative

## Discussion

### Principal Findings

We identified 7 different stakeholder-relevant problem categories (“technology,” “data,” “user,” “studies,” “ethics,” “law,” and “general”). While “technology” included problems that are directly related to the technology of AI-based CDSSs themselves, the other problem categories concerned the environment and the circumstances in which the systems are conceptualized, developed, studied, and used.

While our systematization is fundamentally new, many of the problems identified were in accordance with the literature on CDSSs. For example, Sutton et al [7] described the following problem areas in the context of CDSSs: “fragmented workflows,” “alert fatigue and inappropriate alerts,” “impact on user skill,” “dependence on computer literacy,” “system and content maintenance,” “operational impact of poor data quality and incorrect content,” “lack of transportability and interoperability,” and “financial challenges.” Similar results were obtained in a review of medication-related CDSSs. Most reported factors influencing the acceptance of such systems were (a lack of) “usefulness,” “relevance” of information, “ease of use,” and “efficiency” [17].

While these problems were valid for CDSSs in general, our study showed that stakeholders also saw problems that are especially attributable to the integration of AI into these systems (eg, “generalizability,” “black-box character,” and “perception of AI”).

Challenges of AI in health and medicine have been described, for example, by Rajpurkar et al [18]. They found problems similar to those identified in our research. Challenges were identified in “implementation,” “accountability,” and “fairness.” Implementation challenges included model trust and data limitations; accountability challenges stemmed from regulations and responsibilities; and, finally, fairness challenges comprised ethical data use as well as equity and bias.

In the German context in which the study took place, the Ethics Committee of the German Medical Association provides a profound overview on ethical and legal problem areas in the field of AI. Ethical aspects are “trust and trustworthiness,” “responsibility,” “autonomy,” “communication and empathy,” “medical expertise,” “risk of discrimination,” and “data sovereignty and privacy.” Legal aspects include “regulation of medical devices,” “obligations in the context of examination or treatment methods,” “duties of care of physicians,” “patient information and consent,” “liability,” and “data protection and confidentiality” [19].

In addition to individual problems of CDSSs as well as AI, there is some research on factors influencing the acceptance of AI-based CDSSs. Factors described in this context include concern of system failure, overtrusting of the systems, locality of the systems, and an intuitive user interface [20]. Knop et al [21] listed further characteristics and human factors that influence and shape the relationship and collaboration between AI-based CDSSs and humans. Technological characteristics include “training data quality,” “performance,” “explainability or transparency,” and “adapted output or adaptability.” Human factors comprise “medical expertise,” “technological expertise,” “personality,” “cognitive biases,” and “trust” [21]. While technological characteristics included a part of the problems that we identified and categorized under “technology” and “data,” we included human factors under the category “user.”

The combination of AI and CDSSs can increase the relevance of individual problems. While traditional CDSSs typically provide transparent and understandable reasoning, the black-box nature of AI-based CDSSs can make it difficult to build trust in these systems. If, for example, the causes of alarms are not clear, this could lead to them being ignored. On the other hand, the need for scrutinizing proposed decisions is even more important in AI-based CDSSs. As the reason for a decision is not comprehensible and relies on statistics, physicians should be even more sensitized to automation bias to uncover wrong decisions.

In the context of AI-based CDSSs, a special focus during development should be placed on the creation of the data basis. As the systems learn from these data, there is a threat that wrong decisions will emerge if the data are inadequate.

A problem that arises specifically with AI-based CDSSs is liability for incorrect decisions. As the systems themselves cannot be held liable, it is conceivable that either the physicians using them or the vendors or health care organizations could be held liable [22]. However, it should be noted that there is no easy way of understanding black-box decisions, and therefore, the responsibility of physicians is not clear.

While the problems identified in our research are in accordance with the literature, it should be emphasized that some stakeholders saw certain topics as problems, whereas others did not. One example is “loss of knowledge.” While one caregiver representative mentioned that it would be a problem, another stated that the systems are an upgrade for human decision-making instead of leading to loss of knowledge. Another such example was the problem of executing untested recommendations (automation bias). While most of the stakeholders saw a problem here, one stakeholder emphasized that, if the reliability of the systems was close to 100% in the future, it would no longer be a problem. One interviewee emphasized that, in general, he would rather refer to them as “to-dos” than as problems.

To increase physicians’ acceptance of CDSSs, Khairat et al [23] proposed 2 different models. On the one hand, physicians should be included in the design process by examining user needs and expectations as well as the usability of prototypic designs. On the other hand, it is important for physicians to include the CDSS as a component of their decisions while maintaining professional autonomy. These approaches should also be pursued in the context of AI-based CDSSs.

Even if most problems of AI-based CDSSs were already known from CDSSs or AI as individual technologies, our investigation shows that they are still relevant. Our work in particular makes a contribution by compiling and systematizing the problems in the context of AI-based CDSSs. This provides an ideal starting point for further investigation of individual problem categories or manifestations.

### Implications

AI-based CDSSs can lead to an improved physician performance and better medical outcomes [24]. Nevertheless, we identified several problems that are in accordance with the literature [7,18,19]. These should be solved as, by addressing errors, the potential of AI to improve the future of medicine can be realized [18].

Even if there are regulations for CDSSs, such as the 21st Century Cures Act in the United States and the Medical Device Regulation in the European Union, not all problems regarding this technology are covered [25]. McKee and Wouters [8] identified five challenges for regulators in the context of AI: (1) the AI application is only one part of a complex clinical system, (2) the training process may incorporate existing values and biases, (3) the performance will change over time using ML, (4) there might be conflicts with data protection legislation, and (5) gathering vast quantities of data might raise issues of privacy.

Given the heterogeneity of the problems in the context of AI-based CDSSs, one cannot assume a definite solution to be available. While many problems emerge from the technology behind AI-based CDSSs, there are other problems emerging from the environment in which the systems should be used. Therefore, existing risks cannot be solved only on the technical level but require interdisciplinary effort. To conceptualize valuable systems, clear communication between IT professionals (developers) and health care practitioners (users) during development is needed [26].

Even if future systems can reliably derive decisions from databases, the treating physician should not be left out. Some problems, such as lack of implicit knowledge, dehumanization, and most of the ethical problems or the (medical) context of the data, cannot be sufficiently considered by the systems. Therefore, to achieve the best outcome for all actors within the health care system, the goal should be to combine the capabilities and strengths of AI and human intelligence [12,27]. To do so, it is necessary to identify the attitudes of relevant stakeholders toward the technology and its use. Such an approach was adopted by Laï et al [28]. In an interview study with 40 French stakeholders, they found that views on AI were very heterogeneous and that only cooperation between the involved parties would lead to the development of satisfactory AI tools.

As mentioned in the Methods section, this work is part of a research project—KI@work. The results of this interview study together with the results of a scoping review and focus groups with physicians and carers will be used to conceptualize a quantitative survey for intensive care physicians. Thereby, the relevance of individual problems from the point of view of physicians might be determined more precisely.

### Limitations

Given the novelty of the technology of AI-based CDSSs, we conducted interviews to explore problems and barriers from the point of view of German stakeholders. Due to the qualitative methodology, we interviewed only representatives of each stakeholder group. Therefore, the results are not necessarily representative and should be interpreted with caution. Thus, the findings of this study should be seen as preliminary evidence to guide further qualitative or quantitative research rather than as definitive conclusions.

There are 2 further limitations of this study that should be mentioned. First, we did not calculate any agreement rate or other measurements regarding coding discrepancies. However, quality assurance was conducted via the internal workshop, which aimed to systematize the identified problems and barriers. Second, as the expert interviews took place in Germany, the interviews were conducted in German. Thus, the statements included in this paper were translated. After a first translation, it was checked within the consortium whether the content of the statements had been changed. Upon reasonable request, the original anonymized citations are available from the authors.

In this study, we aimed to obtain a comprehensive overview of problems and barriers from the point of view of different stakeholders. Nevertheless, we had a slight overrepresentation of care professionals. As the views of patients compared to clinicians differ regarding certain aspects, such as trust [29], further studies should explicitly focus on physicians and patients as key stakeholders of AI-based CDSSs.

As we interviewed German individuals and the German system has specific problems, such as low digitalization in health care [30-32], strict regulation regarding the handling of data [33], and a severe shortage of physicians and caregivers [34], the relevance and transferability of the problems to other health care systems should always be critically scrutinized.

### Conclusions

Experts see several problems within the context of AI-based CDSSs. Problems concerned the technology itself as well as the context in which the systems are developed and used. In total, we identified 7 different problem categories, namely, “technology,” “data,” “user,” “studies,” “ethics,” “law,” and “general.” To guarantee a sustainable, safe, and effective integration of AI-based CDSSs, the problems identified in this study should be considered when developing and using these systems. Furthermore, problems that focus on regulation should also be taken into account by policy makers. As this study followed an explorative qualitative design, the results should be further investigated in additional qualitative and quantitative studies.

## Appendix

Multimedia Appendix 1 Interview guidelines.

Multimedia Appendix 2 Systematization of all identified problems.

## Abbreviations

AI: artificial intelligence
CDSS: clinical decision support system
COREQ: Consolidated Criteria for Reporting Qualitative Research
ML: machine learning
